# Predictive Value of Inflammatory Markers NLR, PLR, APRI, SII, and Liver Function Tests in Systemic Inflammatory Response Syndrome Detection in Full-Term Newborns

**DOI:** 10.3390/children11050593

**Published:** 2024-05-14

**Authors:** Manuela Pantea, Daniela Iacob, Mirabela Dima, Mihaela Prodan, Oana Belei, Rodica Anamaria Negrean, Adrian Cosmin Ilie

**Affiliations:** 1 Department of Neonatology, “Victor Babes” University of Medicine and Pharmacy Timisoara, 300041 Timisoara, Romania; manuela.pantea@umft.ro (M.P.); iacob.daniela@umft.ro (D.I.); mirabela.dima@umft.ro (M.D.); 2 Doctoral School, “Victor Babes” University of Medicine and Pharmacy Timisoara, 300041 Timisoara, Romania; mihaela.prodan@umft.ro; 3 First Pediatric Clinic, Disturbances of Growth and Development on Children Research Center, “Victor Babes” University of Medicine and Pharmacy Timisoara, 300041 Timisoara, Romania; belei.oana@umft.ro; 4 Third Pediatric Clinic, “Louis Turcanu” Emergency Hospital for Children, 300011 Timisoara, Romania; 5 Department of Preclinical Disciplines, Faculty of Medicine and Pharmacy, University of Oradea, 410073 Oradea, Romania; 6 Department III Functional Sciences, Division of Public Health and Management, “Victor Babes” University of Medicine and Pharmacy Timisoara, 300041 Timisoara, Romania; ilie.adrian@umft.ro

**Keywords:** inflammatory markers, risk analysis, neonatology, pediatrics

## Abstract

Systemic Inflammatory Response Syndrome (SIRS) is associated with significant morbidity and mortality in full-term newborns. This study aimed to evaluate the predictive value of the Neutrophil-to-Lymphocyte Ratio (NLR), Derived Neutrophil-to-Lymphocyte Ratio (dNLR), Platelet-to-Lymphocyte Ratio (PLR), Neutrophil, Lymphocyte, and Platelet Ratio (NLPR), AST-to-Platelet Ratio Index (APRI), and Systemic Immune–Inflammation Index (SII) in identifying the risk for SIRS development in full-term newborns. Conducted between January 2023 and January 2024, this observational cohort study compared full-term newborns diagnosed with SIRS with newborns without SIRS, measuring the inflammatory markers within the first day of life and three days post-birth. The study included 229 newborns, 81 with SIRS and 148 controls without SIRS. Statistically significant differences were observed in NLR (3.81 vs. 2.20, *p* < 0.0001), PLR (68.12 vs. 52.30, *p* < 0.0001), and liver enzymes (AST 40.96 U/L vs. 31.58 U/L, ALT 34.66 U/L vs. 22.46 U/L, both *p* < 0.0001) between the groups. The NLPR demonstrated substantial diagnostic value, with a sensitivity of 78.36% and specificity of 83.52% at 72 h (*p* < 0.0001). Regression analysis highlighted that the NLPR and SII were strongly predictive of SIRS, with the NLPR showing over three-times higher SIRS risk (HR 3.29, *p* < 0.0001) and SII indicating nearly 3.5 times the risk (HR 3.47, *p* < 0.0001). The NLPR, APRI, and SII showed similar prediction values to CRP levels measured on the first and third days of life (HR 3.16). Inflammatory markers like NLR, PLR, and systemic indices such as NLPR and SII, alongside liver function tests, are significant predictors of SIRS in full-term newborns. These findings support the integration of these markers into routine neonatal care, allowing for early identification and potentially improved management of newborns at risk for SIRS, thereby enhancing clinical outcomes.

## 1. Introduction

The neonatal period, encompassing the first 28 days of life, is a critical phase for newborns, laying the foundation for long-term health and development [1,2]. During this time, neonates are highly susceptible to various challenges, including infections or metabolic changes which can trigger systemic inflammatory responses [3,4]. Systemic Inflammatory Response Syndrome (SIRS) in newborns, while not solely caused by infection, can result from bacterial, viral, or fungal infections, as well as non-infectious processes such as trauma or ischemia [5]. SIRS is characterized by a series of clinical signs including fever or hypothermia, tachycardia, tachypnea, and an abnormal white blood cell count [6]. The condition is a concern due to its potential to escalate into more severe complications like sepsis, multiple organ dysfunction syndrome (MODS), or even death if not promptly recognized and treated [7,8].

Globally, neonatal infections, including those leading to SIRS and sepsis, are a significant cause of morbidity and mortality, particularly in low- and middle-income countries [9,10]. According to the World Health Organization (WHO), nearly 30% of neonatal deaths are due to infections [11,12]. Furthermore, studies have found that the incidence of neonatal sepsis ranges widely, from 1 to 5 per 1000 live births in developed countries to as high as 50 per 1000 live births in parts of Asia and Africa, suggesting the impact of resource availability, maternal health, access to healthcare, and neonatal care practices on the outcomes of newborns [13,14].

In full-term newborns, the incidence of SIRS and subsequent sepsis is lower compared to preterm infants, but it remains a significant concern [15]. Full-term newborns who develop SIRS are often those who have been exposed to risk factors such as maternal infections, prolonged rupture of membranes, or complications during delivery [16]. The presence of SIRS in this population is particularly challenging to diagnose due to the non-specific nature of its clinical presentation, which can overlap with normal neonatal physiological responses or other benign conditions [17].

The adverse outcomes associated with SIRS and neonatal sepsis include prolonged hospitalization, increased need for supportive care, and in severe cases, long-term developmental delays or impairments [18]. Moreover, the economic burden on healthcare systems and families due to neonatal SIRS and sepsis is substantial, with costs accruing from extended hospital stays, intensive care, and long-term management of sequelae [19]. Thus, inflammatory markers such as the Neutrophil-to-Lymphocyte Ratio (NLR) and Platelet-to-Lymphocyte Ratio (PLR) have emerged as potential predictive tools in this context. Studies in adults have established these markers as valuable in predicting the severity and outcome of various inflammatory conditions.

The hypothesis of the current study states that elevated levels of NLR and PLR, coupled with alterations in liver function tests, are significant predictors of SIRS in this population. Consequently, the objectives are twofold: firstly, to validate the predictive value of NLR and PLR in the context of neonatal SIRS and, secondly, to assess the correlation between liver function parameters and the severity of SIRS in full-term newborns, thereby contributing to better clinical management and outcomes.

## 2. Materials and Methods

### 2.1. Design and Ethics

This observational cohort study, spanning January 2023 to January 2024, was conducted in the Neonatal Intensive Care Unit (NICU) of the “Pius Brinzeu” hospital, focusing on and comparing full-term newborns that developed SIRS with those who did not, serving as a control group. Ethical adherence was essential prior to study onset, with the study protocol receiving approval from the hospital’s Ethical Committee for Scientific Research and aligning with the 1964 Helsinki Declaration principles.

Prior to data collection, parents or legal guardians of all neonates were fully briefed about the study’s purpose, benefits, and risks, ensuring informed consent was obtained, thereby guaranteeing voluntary participation. All personal and health information collected was anonymized and securely stored, accessible only to authorized members of the research team, thus upholding the privacy and confidentiality of the participants and maintaining the highest ethical standards throughout the study.

### 2.2. Patients’ Inclusion

The inclusion criteria for this study were carefully defined to ensure a focused and relevant participant group: (1) full-term newborns, classified as those born at or after 37 weeks of gestational age and (2) newborns for whom comprehensive inflammatory marker and liver function tests could be conducted, specifically including measurements necessary to determine the NLR and PLR and key liver function parameters. The purpose behind these criteria was to create a homogeneous group of full-term newborns whose health status could accurately reflect the predictive value of these markers for SIRS.

Exclusion criteria were delineated to maintain the study’s methodological rigor: (1) newborns with significant congenital anomalies that could independently influence the outcome measures of interest, such as those affecting the heart, lungs, or brain, were excluded due to the potential confounding effects on the study’s primary outcomes; (2) infants diagnosed with genetic syndromes, considering the intricate relationship between genetic conditions and neonatal health, which could skew the interpretation of the inflammatory and liver function markers; (3) cases where the neonate succumbed during the study period were excluded to ensure the analysis reflected outcomes of newborns surviving beyond the neonatal phase; (4) any instance where informed consent was not obtained from the parents or legal guardians was automatically excluded, adhering to the ethical standards governing research with human subjects.

For the diagnosis of SIRS in the study’s context, existing guidelines were considered [20], requiring the presence of at least two of the following: a core body temperature of <36 °C (indicative of hypothermia) or >38 °C (fever); a heart rate exceeding 160 beats per minute for newborns up to 1 week old or more than 150 beats per minute for those aged 1 to 4 weeks; a respiratory rate greater than 20 breaths per minute or necessitating mechanical ventilation not attributable to congenital anomalies; and an abnormal white blood cell count, either below 5000 cells/mm^3^ or above 15,000 cells/mm^3^, or having more than 10% immature neutrophils (band forms). These criteria allowed for the identification of neonates experiencing SIRS, enabling a detailed analysis of the proposed predictive markers within this population.

### 2.3. Patient Management and Study Variables

For this study, several blood analyses were performed to evaluate the predictive markers of interest for SIRS in full-term newborns. The complete blood count (CBC), crucial for determining the NLR and PLR, utilized a Sysmex XN-550 automated hematology analyzer, provided by Sysmex Corporation, Kobe, Japan. This process required collecting 1 mL of peripheral venous blood from each newborn into tubes containing Ethylenediaminetetraacetic acid (EDTA).

Liver function tests, essential for assessing the health status of the liver in these newborns, involved measurements of Aspartate Aminotransferase (AST) and Alanine Aminotransferase (ALT), among other parameters. These tests were conducted using high-precision biochemical analyzers that employ spectrophotometric methods for accurate quantification. Additionally, C-reactive protein (CRP) levels, an acute-phase reactant indicative of inflammation, were determined using a Cobas Integra 400 Plus or Cobas e411 analyzer from Roche Diagnostics GmbH, Mannheim, Germany. For CRP and liver function tests, 2 mL of peripheral venous blood was collected in tubes designed with a separator gel to facilitate serum extraction.

Blood samples were collected at two postnatal time points to monitor dynamic changes in the inflammatory and liver function markers. The first collection occurred within the initial hours after birth, establishing baseline levels for all markers. The second collection took place at 72 h after birth, aligning with the critical window for identifying significant shifts in marker levels that could signal the onset of SIRS.

The analytical part of the study involved calculating ratios that reflect the balance of immune responses, potentially indicating the development of SIRS. The NLR was derived by dividing the absolute neutrophil count by the absolute lymphocyte count. Similarly, the PLR was calculated by dividing the absolute platelet count by the absolute lymphocyte count. The dNLR is calculated by dividing the neutrophil count by the difference between the total white cell count and the neutrophil count. The PLR is the ratio of platelets to lymphocytes, both derived from complete blood counts. The NLPR involves the relationship between neutrophils and platelets, while APRI, the AST-to-Platelet Ratio Index, uses the patient’s AST level and platelet count. The SII, or Systemic Immune–Inflammation Index, combines platelet, neutrophil, and lymphocyte counts into a single ratio.

In the current study, the management of pediatric SIRS or sepsis followed established protocols. Broad-spectrum antibiotics were administered within one hour of diagnosis, and oxygen therapy was used to maintain saturation above 94%. Mechanical ventilation was applied as needed, with fluid resuscitation to ensure hemodynamic stability, supplemented by vasoactive drugs if necessary. Continuous monitoring of vital signs and regular lab assessments guided treatment adjustments. Nutritional support and prompt source control of infection, including surgical interventions, were also integral to the management strategy.

### 2.4. Statistical Analysis

Data management and statistical analyses for this study were performed using the statistical software SPSS version 26.0 (SPSS Inc., Chicago, IL, USA). Continuous variables, including the values for inflammatory markers and liver function tests, were summarized as mean ± standard deviation (SD). Categorical variables, such as the presence or absence of SIRS, were presented as frequencies and percentages.

For comparing continuous variables between the two groups (newborns with SIRS versus those without), the Student’s *t*-test was employed. The Chi-square test was utilized for comparing categorical variables. To ascertain the predictive value of the inflammatory markers (NLR and PLR) and liver function tests for SIRS, the analysis included calculations of the best cutoff values, sensitivity, specificity, and Area Under the Curve (AUC) derived from the Receiver Operating Characteristic (ROC) curves. Moreover, a regression analysis, adjusting for potential confounders such as birth weight and APGAR score, was conducted to identify the risk of developing SIRS based on the laboratory parameters exceeding the established cutoff values. Statistical significance was determined by a *p*-value of less than 0.05.

Sample size calculation and study power were determined based on a convenience sampling method, suggesting the differences in NLR and PLR values between newborns with and without SIRS. Assuming an expected mean difference in NLR values between the two groups of 1.5 units with a standard deviation of 1.5 units derived from the previous literature [21] and aiming for a power of 80% to detect this difference at a 5% significance level and 5% margin of error, the required sample size was calculated at a minimum of 139 patients.

## 3. Results

A total of 81 newborns who developed SIRS were included in the study, as well as 148 who did not develop SIRS. The mean gestational age was similar between the two groups, with the SIRS group averaging 39.07 weeks and the No SIRS group 39.22 weeks, resulting in no significant difference (*p* = 0.3358). Gestational weight also showed no significant differences between the groups. The SIRS group had an average weight of 3303.44 g compared to 3394.56 g in the No SIRS group (*p* = 0.1005). When classifying weights into low, normal, and high, the differences remained statistically insignificant (*p* = 0.0949).

Gender distribution between male and female newborns in both groups was also statistically comparable (*p* = 0.5097). The APGAR score, however, presented a significant difference. The SIRS group had a mean APGAR score lower than the No SIRS group, with statistically significant differences both in mean scores and distribution above and below 7 (*p* < 0.0001). The presence of Group B Streptococcus-positive culture and the incidence of cesarean births were higher in the SIRS group, though these did not reach statistical significance (*p* = 0.1003 for GBS and *p* = 0.0636 for cesarean births), as presented in Table 1.

The average pH values did not differ significantly between the two groups, with the SIRS group at 7.35 and the No SIRS group at 7.34 (*p* = 0.3271), suggesting similar acid–base balance at birth. Similarly, the partial pressures of carbon dioxide (pCO_2_) and oxygen (pO_2_) levels were comparable between groups, with *p*-values of 0.0751 and 0.6519, respectively, indicating no significant respiratory differences at birth. However, lactate levels were significantly higher in the SIRS group (3.42 mmol/L) compared to the No SIRS group (2.67 mmol/L), with a *p*-value of 0.0001. Elevated lactate can be a marker of metabolic stress or hypoxia, which is consistent with the pathological processes associated with SIRS.

Significant differences were also observed in white blood cell count (WBC), with the SIRS group having a higher mean WBC of 10.58 × 10^9^/L compared to 8.57 × 10^9^/L in the No SIRS group (*p* < 0.0001). Neutrophils, lymphocytes, and platelet counts were significantly different, with the SIRS group showing higher counts of neutrophils and lymphocytes and lower platelet counts compared to the No SIRS group, all with *p*-values < 0.0001. Markers of inflammation and tissue damage such as CRP and LDH were significantly higher in the SIRS group (CRP: 10.32 mg/L, LDH: 245.05 U/L) compared to the No SIRS group (CRP: 5.07 mg/L, LDH: 199.31 U/L), with *p*-values < 0.0001 for both.

Liver enzymes AST and ALT were significantly elevated in the SIRS group, with AST levels at 40.96 U/L compared to 31.58 U/L in the No SIRS group and ALT levels at 34.66 U/L versus 22.46 U/L, respectively (both *p* < 0.0001). Additionally, inflammatory ratios such as the NLR, PLR, and SII were markedly higher in the SIRS group. Specifically, NLR was 3.81 in the SIRS group compared to 2.20 in the No SIRS group, PLR was 68.12 versus 52.30, and SII was 278.49 versus 225.80 (all *p* < 0.0001), as described in Table 2.

Firstly, blood gas parameters such as pH and partial pressures of carbon dioxide (pCO_2_) and oxygen (pO_2_) did not show significant differences between the SIRS and No SIRS groups, with *p*-values of 0.1795, 0.1530, and 0.1160, respectively. Lactate levels, which are indicative of metabolic stress, remained significantly higher in the SIRS group (3.16 mmol/L) compared to the No SIRS group (2.49 mmol/L), with a *p*-value of <0.0001. White blood cell count, neutrophils, and lymphocytes were significantly higher in the SIRS group compared to the No SIRS group (*p* < 0.0001 for all), suggesting sustained immune activation. Platelet counts were significantly lower in the SIRS group (231.57 × 10^9^/L vs. 292.56 × 10^9^/L in the No SIRS group; *p* < 0.0001).

Elevations in biomarkers of inflammation and cell damage were prominent in the SIRS group. C-reactive protein (CRP) and lactate dehydrogenase (LDH) levels were markedly higher in the SIRS group (CRP: 10.18 mg/L; LDH: 452.38 U/L) compared to the No SIRS group (CRP: 6.99 mg/L; LDH: 221.82 U/L), both with *p*-values < 0.0001.

Inflammatory indices including the NLR, dNLR, PLR, NLPR, APRI, and SII were all significantly elevated in the SIRS group three days after birth, indicating a persistent inflammatory response. Specifically, the NLR was recorded at 3.28 in the SIRS group compared to 2.05 in the No SIRS group, the dNLR at 2.56 versus 1.90, PLR at 122.94 versus 81.25, and the SII was remarkably higher at 317.22 compared to 192.13 (all *p* < 0.0001). Additionally, significant elevations were noted in liver enzymes, with AST at 69.34 U/L and ALT at 52.67 U/L in the SIRS group, both significantly higher than in the No SIRS group (28.18 U/L and 22.09 U/L respectively; *p* < 0.0001). Other markers, such as lactate (3.16 mmol/L vs. 2.49 mmol/L), white blood cells (12.65 × 10^9^/L vs. 9.14 × 10^9^/L), and lactate dehydrogenase (LDH; 452.38 U/L compared to 221.82 U/L), were higher in the SIRs group compared to the No SIRS group (all *p* < 0.0001), as presented in Table 3.

On the first day of life, the NLR illustrates a clear trend, with the lowest average NLR observed in the No SIRS group (2.20), increasing in the viral infection group (3.66) and reaching the highest in the bacterial infection group (4.25). Similarly, the dNLR escalates from 1.98 in the No SIRS group to 2.85 in the viral infection group and further to 3.32 in the bacterial infection group, suggesting a heightened systemic inflammatory response associated with bacterial infections. The PLR also varies significantly, starting at 52.30 in the non-infected group, increasing to 77.20 in the viral infection group, and peaking at 88.45 in the bacterial infection group. The NLPR and the APRI similarly show the highest values in the bacterial infection group, pointing to robust inflammatory and hepatic involvement in these cases. Similarly, CRP values were significantly higher in the viral infection and bacterial infection groups, with a slight increase from the first to third days of life. The SII progressively increases from 225.80 in the non-infected group to 310.21 in the viral infection group and 365.37 in the bacterial group, as presented in Table 4.

For the laboratory parameters assessed at 24 h from birth, the Neutrophil-to-Lymphocyte Ratio (NLR) presented a cutoff value of 7.82, achieving sensitivity and specificity rates of 64.43% and 65.29%, respectively, although not statistically significant (*p* = 0.088). The Derived Neutrophil-to-Lymphocyte Ratio (dNLR) showed a cutoff value of 4.98, with higher sensitivity and specificity of 71.67% and 68.14%, respectively (*p* = 0.027). The Platelet-to-Lymphocyte Ratio (PLR) was established at a cutoff of 212, with sensitivity and specificity of 62.19% and 74.88%, respectively (*p* = 0.009). Additionally, the Neutrophil, Lymphocyte, and Platelet Ratio (NLPR) demonstrated a cutoff of 0.41, with sensitivity and specificity rates of 69.34% and 79.62%, respectively, showing strong predictive capability (*p* < 0.001). The AST-to-Platelet Ratio Index (APRI) displayed a cutoff of 1.51, with sensitivity of 54.97% and notably high specificity of 82.45% (*p* = 0.037). The Systemic Immune–Inflammation Index (SII) set at 496 showed sensitivity and specificity of 72.13% and 69.26%, respectively (*p* = 0.001).

**Figure 1:**
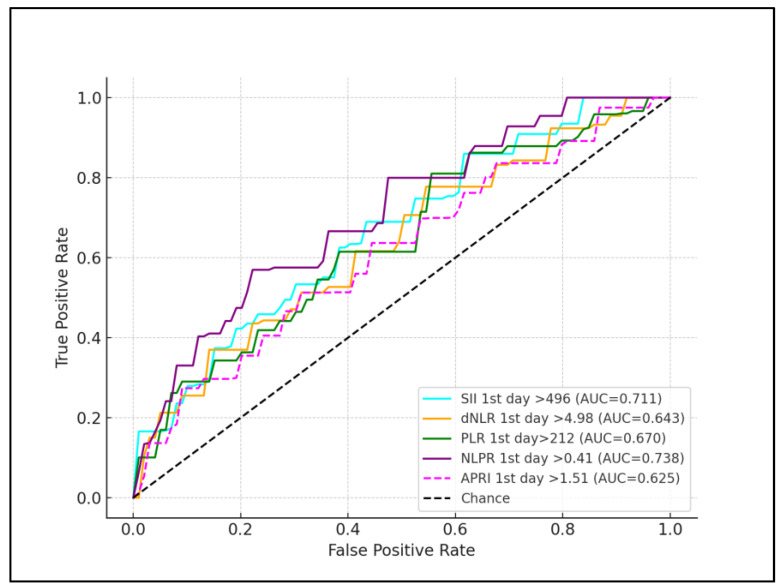
ROC curve for SIRS predictors during the first day of life.

**Figure 2:**
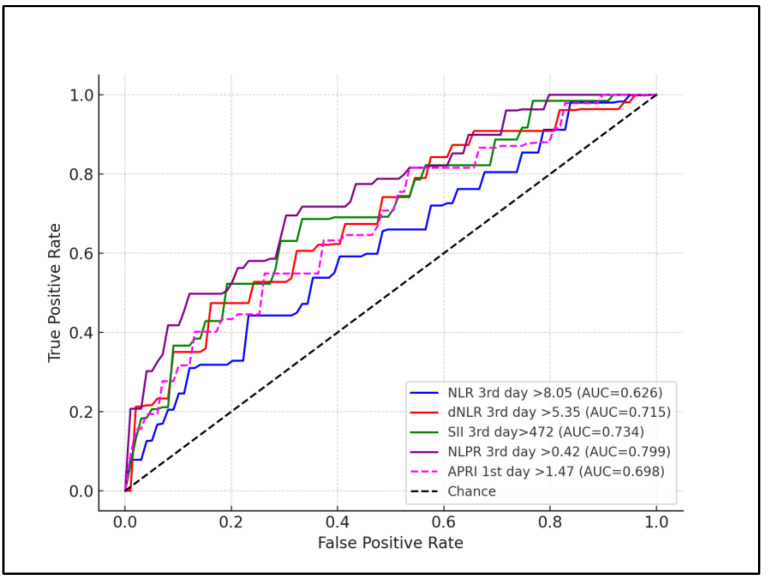
ROC curve for SIRS predictors at three days after birth.

Regarding assessments performed at 72 h after birth, the Neutrophil-to-Lymphocyte Ratio (NLR) had a slightly adjusted cutoff of 8.05, showing increased sensitivity and specificity of 70.22% and 66.89%, respectively (*p* = 0.023). The Derived Neutrophil-to-Lymphocyte Ratio (dNLR) displayed a cutoff of 5.35, with enhanced sensitivity and specificity of 69.58% and 75.41%, respectively (*p* = 0.001). The Platelet-to-Lymphocyte Ratio (PLR) demonstrated a cutoff of 325 but with lower sensitivity and specificity of 63.11% and 66.04%, respectively, not showing significant predictive strength (*p* = 0.134). The Neutrophil, Lymphocyte, and Platelet Ratio (NLPR) for the third day showed a cutoff of 0.42, with high sensitivity and specificity of 78.36% and 83.52%, respectively, reinforcing its diagnostic utility (*p* = 0.011). The AST-to-Platelet Ratio Index (APRI) on the third day had a cutoff of 1.47, with sensitivity and specificity of 59.78% and 81.27%, respectively (*p* = 0.002). The Systemic Immune–Inflammation Index (SII) cutoff was 472, showing high sensitivity of 77.13% and specificity of 71.26% (*p* < 0.001). CRP also showed a similar sensitivity and specificity with the other inflammatory scores, having the second highest AUC for first day of life measurement (AUC = 0.668), and the third highest AUC for third day of life measurements (AUC = 0.719), as presented in Table 5, Figure 1 and Figure 2.

The Neutrophil-to-Lymphocyte Ratio showed a hazard ratio of 1.29, suggesting a 29% increased risk of developing SIRS when the NLR is above the cutoff, although this association was not statistically significant (*p* = 0.0941). In contrast, the dNLR exhibited a much stronger association, with a hazard ratio of 2.13, indicating that newborns with dNLR values above the cutoff are more than twice as likely to develop SIRS compared to those below the cutoff, and this finding was statistically significant (*p* = 0.0001). The PLR had a 44% increased risk of SIRS development when above the cutoff, although the result was not statistically significant.

The NLPR demonstrated a very strong predictive value with a hazard ratio of 3.29, indicating a more than threefold increase in the risk of SIRS (*p* < 0.0001). Similarly, the AST-to-Platelet Ratio Index (APRI) showed a significant association with a hazard ratio of 3.03, indicating that values above the cutoff more than triple the risk of developing SIRS (*p* < 0.0001). The SII score also indicated a highly significant and strong predictive association with SIRS development, with a hazard ratio of 3.47 (*p* < 0.0001). Lastly, CRP showed a highly significant and strong predictive association with SIRS development, with a hazard ratio of 3.16 (*p* < 0.0001), having a similar prediction value as the NLPR (HR = 3.29), APRI (HR = 3.03), and SII (HR = 3.47), as described in Table 6.

## 4. Discussion

### 4.1. Literature Findings

The findings of this study highlight the complex interplay of various biochemical and cellular parameters in the early detection of SIRS in full-term newborns. One of the noteworthy observations from the results was the significant elevation in markers like lactate, C-reactive protein, and lactate dehydrogenase in the SIRS group, which suggest a robust metabolic and inflammatory response. These findings corroborate the hypothesis that SIRS is associated with physiological stress and tissue damage, which are detectable through these biomarkers. The elevation in lactate, a known marker of hypoxia and metabolic distress, further underscores the severity of SIRS and potentially guides the clinical management of these infants.

The study also found significant differences in inflammatory indices such as the Neutrophil-to-Lymphocyte Ratio, Platelet-to-Lymphocyte Ratio, and the Systemic Immune–Inflammation Index, which were all higher in the SIRS group. This indicates an active inflammatory process consistent with the pathophysiology of SIRS. These indices, particularly SII, which showed the most substantial differences, could serve as practical biomarkers for early SIRS detection. This is particularly relevant in clinical settings where rapid decision-making is crucial for effective management and treatment of affected neonates.

Liver function tests, including AST and ALT, were notably higher in the SIRS group, which might suggest hepatic involvement or stress as part of the systemic response to inflammation. The implication of liver function tests in SIRS has not been extensively studied in neonates and presents an area for further research. Understanding the role of hepatic response in SIRS could enhance our comprehension of the disease’s systemic nature and potentially prompt the development of targeted therapies that address multiple organ systems.

Particularly, markers like the dNLR and NLPR showed strong associations with the development of SIRS, as reflected in their high hazard ratios. These results underscore the potential of using a combination of simple blood tests to develop a predictive model for early identification of newborns at risk for SIRS. This could fundamentally change the approach to monitoring and early intervention in neonates suspected of developing this serious condition, possibly reducing morbidity and improving outcomes.

Other studies underlined the diagnostic potential of the NLR across different pediatric conditions, complementing our findings on its predictive value for SIRS in full-term newborns. Karabulut et al. [22] demonstrated the NLR’s high sensitivity (88%) and specificity (84%) in detecting early-onset neonatal sepsis with an impressive AUC of 0.891, reinforcing the NLR’s utility in neonatal settings. Similarly, Zhong et al. [23] highlighted the NLR’s efficacy in predicting severe pediatric sepsis, achieving an AUC of 0.715, and noted improved outcomes when combined with other biomarkers. These studies affirm the robustness of the NLR as a reliable inflammatory marker, supporting its integration into clinical protocols for early detection and management of neonatal and pediatric sepsis, thereby enhancing our study’s relevance and application in neonatal intensive care.

The studies by Li et al. [24] and Arcagok et al. [25] provide compelling evidence for the utility of inflammatory markers in sepsis prediction, which aligns with the findings of our study on SIRS in full-term newborns. Li et al. highlighted that combining the NLR with Sequential Organ Failure Assessment (SOFA) significantly enhances prediction of 28-day mortality in adult sepsis patients, achieving an odds ratio (OR) of 1.455 (95% CI 1.318–1.605) with improved sensitivity and specificity over individual scores. Similarly, Arcagok et al. demonstrated in neonates that the PLR has excellent predictive accuracy for early-onset sepsis, with an AUC of 0.89 to 0.93 and sensitivity of 88.9% to 91.3% at cutoff values ranging from 39.5 to 57.7.

Other research findings provide valuable context for our study’s focus on SIRS in newborns, emphasizing the diagnostic relevance of inflammatory markers across different age groups and settings. Mahmoud et al. [26] reported high specificity and positive predictive value for the NLR and PLR in detecting early-onset neonatal sepsis (NLR 99% specificity, 98% PPV; PLR 73% specificity, 72% PPV), underscoring their utility as reliable indicators of sepsis in neonates. Similarly, Bacarea et al.’s [27] analysis in adult sepsis patients highlighted the prognostic significance of dynamic changes in the NLR and PLR, alongside systemic inflammation markers, reinforcing the potential of these biomarkers to reflect the severity and progression of sepsis.

Similarly relevant in the context of the current study are the results described by Tamelytė et al. [28], who found significant utility in using the Platelet-to-Mean-Platelet-Volume (PLT/MPV) ratio and NLR for early detection of sepsis/bacteremia, with the NLR showing high specificity (99%) and the PLT/MPV ratio distinguishing early-arrival sepsis/bacteremia cases effectively (42.70 ± 8.57 vs. 31.01 ± 8.21, *p* = 0.008). These findings align with our study’s emphasis on the NLR and PLR as key predictors, highlighting their potential to differentiate between bacterial and viral infections effectively. On the other hand, Poggi’s examination of presepsin showed impressive sensitivity (93%) and specificity (91%) in diagnosing early-onset sepsis, suggesting that, like our SIRS markers, presepsin could significantly enhance early sepsis detection and management in neonates [29].

Chang et al.’s meta-analysis highlighted the effectiveness of soluble TREM-1 (sTREM-1) as a biomarker, with its impressive diagnostic and prognostic capabilities demonstrated by high sensitivity (0.95), specificity (0.98), and an area under the SROC curve of 0.99 for neonatal sepsis [30]. These findings suggest that sTREM-1, like our study’s NLR and PLR, could be instrumental in improving outcomes through early sepsis detection. Similarly, Yılmaz Oztorun’s examination of serum uric acid and NLR in late-onset neonatal sepsis reveals that elevated NLR and serum uric acid levels correlate significantly with sepsis, echoing our findings where elevated NLR was a robust predictor for SIRS [31]. The sensitivity (35%) and specificity (95%) of uric acid levels in diagnosing sepsis, though not as high as in Chang et al.’s study, still underscore the potential for these markers to enhance diagnostic accuracy in neonatal care, particularly when combined with clinical assessments. These comparative insights reinforce the importance of integrating a range of biomarkers, including sTREM-1 and uric acid, alongside traditional inflammatory indices to refine the early detection and management of neonatal sepsis and SIRS.

Other studies demonstrated the clinical value of CRP as a predictor of neonatal sepsis. In Xiaojuan Li et al.’s study [32], CRP showed moderate predictive accuracy (AUC = 0.68) for identifying neonatal sepsis. Tiewei Li et al. [33] reported a stronger association, with CAR showing an AUC of 0.74 for predicting sepsis and 0.70 for severe sepsis, indicating its utility as a substantial independent risk factor. Comparatively, our study investigated a broader range of inflammatory markers, where, notably, the NLPR and SII demonstrated significant predictive value, with risk ratios indicating a three to three-and-a-half times higher risk of developing SIRS, comparable to CRP levels measured on the first and third days of life. The high specificity and sensitivity reported for the NLPR (sensitivity of 78.36% and specificity of 83.52% at 72 h) suggest its potential utility in clinical settings.

While CRP and ratios like CRP and CAR provide valuable insights into the risk of neonatal sepsis, the inflammatory markers investigated in the current study offer a broader diagnostic scope by considering multiple physiological parameters. This could enhance the accuracy and timeliness of SIRS diagnosis in newborns, supporting early and targeted intervention. Future perspectives could involve integrating these markers into a prognostic model that also includes clinical variables, potentially increasing predictive accuracy and providing a more comprehensive assessment tool than either clinical variables or biomarkers alone.

### 4.2. Limitations and Future Perspectives

One significant limitation of this study is its observational design, which, while effective for identifying associations, does not establish causality between the elevated inflammatory markers and the development of SIRS in full-term newborns. The study was conducted in a single hospital, which may limit the generalizability of the findings to other settings with different patient demographics or clinical practices. Additionally, the reliance on specific cutoff values for predictive markers may not account for individual variability in immune response among newborns. Furthermore, the exclusion of newborns with significant congenital anomalies or genetic syndromes might have excluded a subset of the population at higher risk for SIRS, potentially skewing the results towards a healthier cohort.

Considering our study focused exclusively on a newborn population, it is important to emphasize that our findings are validated only within this specific demographic, which possesses unique physiological characteristics and immune responses. Moreover, the definition of SIRS varies by age-specific factors. Therefore, while our results provide valuable insights into SIRS in newborns, the applicability of the NLR, dNLR, PLR, NLPR, APRI, and SII as predictors for sepsis to older pediatric populations remains to be determined. Future research should aim to validate these findings in broader pediatric age groups, specifically focusing on children with sepsis.

## 5. Conclusions

The conclusions of this study reinforce the clinical utility of inflammatory markers such as the NLR, PLR, APRI, SII, and liver function tests as significant predictors of SIRS in full-term newborns. The findings highlight that elevated levels of these markers are closely associated with the occurrence of SIRS, suggesting their potential use in neonatal intensive care settings for early diagnosis and intervention. Incorporating these markers into routine neonatal evaluation could enable healthcare providers to identify at-risk newborns promptly, allowing for targeted management strategies that could potentially reduce the morbidity and mortality associated with SIRS. These results advocate for the adoption of these biomarkers in standard neonatal care, improving outcomes through precise and timely medical responses.

## Figures and Tables

**Table 1:** Background characteristics compared between newborns with and without SIRS.

Variables	SIRS Group (*n* = 81)	No SIRS Group (*n* = 148)	*p*-Value
Gestational age (mean ± SD)	39.07 ± 0.95	39.22 ± 1.01	0.3358
Gestational age, *n* (%)			0.1377
Early term (37–38 weeks)	38 (46.91%)	50 (33.78%)	
Full preterm (39–40 weeks)	27 (33.33%)	58 (39.19%)	
Late preterm (>40 weeks)	15 (18.52%)	40 (27.03%)	
Gestational weight (mean ± SD)	3303.44 ± 398.54 g	3394.56 ± 400.36 g	0.1005
Gestational weight, *n* (%)			0.0949
Low (1500–2499 g)	2 (2.47%)	0 (0%)	
Normal (2500–4000 g)	75 (92.59%)	135 (91.22%)	
High (>4000 g)	4 (4.94%)	13 (8.78%)	
Gender, *n* (%)			0.5097
Male	42 (51.85%)	70 (47.30%)	
Female	39 (48.15%)	78 (52.70%)	
APGAR (mean ± SD)	7.28 ± 1.50	8.20 ± 1.25	<0.0001
APGAR, *n* (%)			<0.0001
≤7	37 (45.68%)	24 (16.22%)	
>7	44 (54.32%)	124 (83.78%)	
SIRS cause			
Viral infection	45 (55.56%)	–	
Bacterial infection	36 (44.44%)	–	
GBS-positive culture	10 (12.35%)	9 (6.08%)	0.1003
Cesarean birth	15 (18.52%)	44 (29.73%)	0.0636

SD—Standard Deviation; GBS—Group B Streptococcus; APGAR—Appearance Pulse Grimace Activity and Respiration; SIRS—Systemic Inflammatory Response Syndrome.

**Table 2:** Laboratory parameters measured during the first day of life.

Variables *	SIRS Group (*n* = 81)	No SIRS Group (*n* = 148)	*p*-Value
pH-CO	7.35 ± 0.08	7.34 ± 0.07	0.3271
pCO_2_	38.93 ± 6.95 mmHg	40.63 ± 6.84 mmHg	0.0751
pO_2_	59.90 ± 9.34 mmHg	59.31 ± 9.51 mmHg	0.6519
Lactate	3.42 ± 1.33 mmol/L	2.67 ± 0.91 mmol/L	0.0001
WBC	10.58 ± 3.03 × 10^9^/L	8.57 ± 2.90 × 10^9^/L	<0.0001
Neutrophils	7.55 ± 2.24 × 10^9^/L	5.71 ± 1.91 × 10^9^/L	<0.0001
Lymphocytes	3.85 ± 0.98 × 10^9^/L	3.02 ± 0.99 × 10^9^/L	<0.0001
Platelets	213.46 ± 46.46 × 10^9^/L	252.81 ± 52.20 × 10^9^/L	<0.0001
CRP	10.32 ± 5.82 mg/L	5.07 ± 5.11 mg/L	<0.0001
LDH	245.05 ± 72.45 U/L	199.31 ± 51.12 U/L	<0.0001
CK	96.81 ± 23.97 U/L	98.46 ± 23.04 U/L	0.6100
AST	40.96 ± 8.08 U/L	31.58 ± 9.42 U/L	<0.0001
ALT	34.66 ± 7.63 U/L	22.46 ± 7.69 U/L	<0.0001
NLR	3.81 ± 1.45	2.20 ± 1.07	<0.0001
dNLR	3.30 ± 17.16	1.98 ± 0.50	<0.0001
PLR	68.12 ± 24.95	52.30 ± 22.78	<0.0001
NLPR	0.26 ± 0.18	0.11 ± 0.12	<0.0001
APRI	1.39 ± 0.85	0.88 ± 0.67	<0.0001
SII	278.49 ± 133.15	225.80 ± 89.22	<0.0001

*—Data presented as mean ± SD; SD—Standard Deviation; SIRS—Systemic Inflammatory Response Syndrome; NLR—Neutrophil-to-Lymphocyte Ratio; dNLR—Derived Neutrophil-to-Lymphocyte Ratio; PLR—Platelet-to-Lymphocyte Ratio; NLPR—Neutrophil, Lymphocyte, and Platelet Ratio; APRI—AST-to-Platelet Ratio Index; SII—Systemic Immune–Inflammation Index; pH-CO—Partial Pressure of Carbon Dioxide (Normal Range: 7.35–7.45); pCO_2_—Partial Pressure of Carbon Dioxide (Normal Range: 35–45 mmHg); pO_2_—Partial Pressure of Oxygen (Normal Range: 50–70 mmHg); WBC—White Blood Cells (Normal Range: 5.0–10.0 × 10^9^/L); CRP—C-Reactive Protein (Normal Range: <10 mg/L); LDH—Lactate Dehydrogenase (Normal Range: 135–225 U/L); CK—Creatine Kinase (Normal Range: 52–336 U/L); AST—Aspartate Aminotransferase (Normal Range: 0–40 U/L); ALT—Alanine Aminotransferase (Normal Range: 0–40 U/L).

**Table 3:** Laboratory parameters measured at three days after birth.

Variables *	SIRS Group (*n* = 81)	No SIRS Group (*n* = 148)	*p*-Value
pH-CO	7.37 ± 0.06	7.36 ± 0.05	0.1795
pCO_2_	38.80 ± 5.61	37.75 ± 5.12	0.1530
pO_2_	58.22 ± 9.27	60.33 ± 9.89	0.1160
Lactate	3.16 ± 1.49	2.49 ± 1.05	<0.0001
WBC	12.65 ± 4.04	9.14 ± 2.78	<0.0001
Neutrophils	7.84 ± 3.19	5.42 ± 2.01	<0.0001
Lymphocytes	4.86 ± 0.99	3.09 ± 0.92	<0.0001
Platelets	231.57 ± 52.33	292.56 ± 49.83	<0.0001
CRP	10.18 ± 6.23	6.99 ± 4.82	<0.0001
LDH	452.38 ± 66.99	221.82 ± 45.19	<0.0001
CK	294.32 ± 124.40	160.23 ± 98.42	<0.0001
AST	69.34 ± 9.57	28.18 ± 9.95	<0.0001
ALT	52.67 ± 7.75	22.09 ± 8.52	<0.0001
NLR	3.28 ± 1.61	2.05 ± 0.91	<0.0001
dNLR	2.56 ± 1.11	1.90 ± 0.52	<0.0001
PLR	122.94 ± 37.61	81.25 ± 18.00	<0.0001
NLPR	0.25 ± 0.08	0.14 ± 0.04	<0.0001
APRI	1.46 ± 1.29	0.73 ± 0.82	<0.0001
SII	317.22 ± 151.20	192.13 ± 96.45	<0.0001

*—Data presented as mean ± SD; SD—Standard Deviation; SIRS—Systemic Inflammatory Response Syndrome; NLR—Neutrophil-to-Lymphocyte Ratio; dNLR—Derived Neutrophil-to-Lymphocyte Ratio; PLR—Platelet-to-Lymphocyte Ratio; NLPR—Neutrophil, Lymphocyte, and Platelet Ratio; APRI—AST-to-Platelet Ratio Index; SII—Systemic Inflammation Index; pH-CO—Partial Pressure of Carbon Dioxide (Normal Range: 7.35–7.45); pCO_2_—Partial Pressure of Carbon Dioxide (Normal Range: 35–45 mmHg); pO_2_—Partial Pressure of Oxygen (Normal Range: 50–70 mmHg); WBC—White Blood Cells (Normal Range: 5.0–10.0 × 10^9^/L); CRP—C-Reactive Protein (Normal Range: <10 mg/L); LDH—Lactate Dehydrogenase (Normal Range: 135–225 U/L); CK—Creatine Kinase (Normal Range: 52–336 U/L); AST—Aspartate Aminotransferase (Normal Range: 0–40 U/L); ALT—Alanine Aminotransferase (Normal Range: 0–40 U/L).

**Table 4:** Comparison of inflammation scores between newborns with SIRS caused by viral infection, bacterial infection, and non-infection.

Variables *	No SIRS Group (*n* = 148)	Viral Infection (*n* = 45)	Bacterial Infection (*n* = 36)	*p*-Value
1st day of life				
NLR	2.20 ± 1.07	3.66 ± 1.32	4.25 ± 1.44	<0.0001
dNLR	1.98 ± 0.50	2.85 ± 0.64	3.32 ± 0.68	<0.0001
PLR	52.30 ± 22.78	77.20 ± 25.34	88.45 ± 29.18	<0.0001
NLPR	0.11 ± 0.12	0.17 ± 0.14	0.22 ± 0.15	<0.0001
APRI	0.88 ± 0.67	1.28 ± 0.72	1.69 ± 0.71	<0.0001
SII	225.80 ± 89.22	310.21 ± 95.40	365.37 ± 100.59	<0.0001
CRP	5.07 ± 5.11	9.48 ± 4.16	10.86 ± 5.93	<0.0001
3rd day of life				
NLR	2.05 ± 0.91	3.19 ± 1.55	4.11 ± 1.74	<0.0001
dNLR	1.90 ± 0.52	2.29 ± 1.18	3.24 ± 1.25	<0.0001
PLR	81.25 ± 18.00	117.91 ± 34.56	140.22 ± 42.04	<0.0001
NLPR	0.14 ± 0.04	0.29 ± 0.08	0.30 ± 0.09	<0.0001
APRI	0.73 ± 0.82	1.36 ± 1.37	1.73 ± 1.37	<0.0001
SII	192.13 ± 96.45	306.28 ± 136.2	375.53 ± 165.88	<0.0001
CRP	6.99 ± 4.82	10.25 ± 6.07	11.81 ± 5.62	<0.0001

*—Data presented as mean ± SD; SD—Standard Deviation; SIRS—Systemic Inflammatory Response Syndrome; NLR—Neutrophil-to-Lymphocyte Ratio; dNLR—Derived Neutrophil-to-Lymphocyte Ratio; PLR—Platelet-to-Lymphocyte Ratio; NLPR—Neutrophil, Lymphocyte, and Platelet Ratio; APRI—AST-to-Platelet Ratio Index; SII—Systemic Inflammation Index; CRP—C-reactive Protein.

**Table 5:** Best cutoff values for predicting SIRS development.

Laboratory Parameter	Timeframe	Best Cutoff Value	Sensitivity	Specificity	AUC	*p*-Value
NLR	1st day	7.82	64.43%	65.29%	0.549	0.088
dNLR	1st day	4.98	71.67%	68.14%	0.643	0.027
PLR	1st day	212	62.19%	74.88%	0.670	0.009
NLPR	1st day	0.41	69.34%	79.62%	0.738	<0.001
APRI	1st day	1.51	54.97%	82.45%	0.625	0.037
SII	1st day	496	72.13%	69.26%	0.711	0.001
CRP	1st day	10	71.22%	80.47%	0.668	<0.001
NLR	3rd day	8.05	70.22%	66.89%	0.626	0.023
dNLR	3rd day	5.35	69.58%	75.41%	0.715	0.001
PLR	3rd day	325	63.11%	66.04%	0.530	0.134
NLPR	3rd day	0.42	78.36%	83.52%	0.799	0.011
APRI	3rd day	1.47	59.78%	81.27%	0.698	0.002
SII	3rd day	472	77.13%	71.26%	0.734	<0.001
CRP	3rd day	10	76.41%	82.15%	0.719	<0.001

SIRS—Systemic Inflammatory Response Syndrome; NLR—Neutrophil-to-Lymphocyte Ratio; dNLR—Derived Neutrophil-to-Lymphocyte Ratio; PLR—Platelet-to-Lymphocyte Ratio; NLPR—Neutrophil, Lymphocyte, and Platelet Ratio; APRI—AST-to-Platelet Ratio Index; SII—Systemic Immune–Inflammation Index; CRP—C-reactive Protein.

**Table 6:** Regression analysis for SIRS development during the neonatal period neonates born at term.

Factors above the Best Cutoff	Hazard Ratio	95% CI	*p*-Value
NLR	1.29	0.93–2.29	0.0941
dNLR	2.13	1.27–4.06	0.0001
PLR	1.44	0.99–3.52	0.0588
NLPR	3.29	2.37–6.34	<0.0001
APRI	3.03	1.49–5.67	<0.0001
SII	3.47	2.04–6.36	<0.0001
CRP	3.16	1.14–7.25	<0.001

SIRS—Systemic Inflammatory Response Syndrome; NLR—Neutrophil-to-Lymphocyte Ratio; dNLR—Derived Neutrophil-to-Lymphocyte Ratio; PLR—Platelet-to-Lymphocyte Ratio; NLPR—Neutrophil, Lymphocyte, and Platelet Ratio; APRI—AST-to-Platelet Ratio Index; SII—Systemic Inflammation Index; CI—Confidence Interval; CRP—C-reactive Protein.

## Data Availability

The data presented in this study are available on request from the corresponding author due to ethical reasons.
